# Query

**Published:** 1888-12

**Authors:** 


### QUERY.

When medical men of high reputation, who are acknowledged leaders by virtue of their great attainments as well as of length of service, who are or have been teachers of medicine, who have held high positions of honor in local, State and National medical associations, and who can plead neither poverty nor lack of practice as an excuse for unethical procedures, when such men, ignoring solemn obligations voluntarily assumed, cause not only their names but even their pictures to appear in secular papers, accompanied by laudatory notices extolling their great skill and wide reputation, how can it be expected that the rank and file of the profession, the humble practitioners, who have no lucrative specialties to advertise, will continue to strive to maintain the dignity of their calling, and to be governed by the requirements of the Code of Ethics, of which one section reads as follows:

"It is derogatory to the dignity of the profession to resort to public advertisements or private cards or hand-bills inviting the attention of individuals affected with particular diseases. * * * These are the ordinary practices of empirics, and are highly reprehensible in a regular physician?" We pause for a reply.

### ITEMS.

OVER two hundred cases of sickness, caused by eating cheese, have been reported to the Ohio State Board of Health within a few months, the towns of Urbana and Mansfield contributing fifty cases each. Grave symptoms, even to complete syncope, appeared in some cases, but there were no deaths.

IT is said that Omaha, Neb., has a medical society that has no rules, no officers, no annual dues and meets twice a month at the residence of a member who wants to read a paper. This plan of organization has the merit of extreme simplicity, but we suspect that its results will be often the "go as you please."

THE NEW ORLEANS POLYCLINIC.—This institution, with a full corps of first-class instructors, has been successfully inaugurated. The spring session will open on the first Monday in April, 1889, and continue two full months. The Charity Hospital affords abundance of clinical material. Address Dr. J. H. Bemiss, President, for particulars.

"HOW MUCH DO I OWE YOU?"—This question is often asked the physician by his patient and frequently met with the response, "I will post up my books and let you know."

By a clever and ingenious arrangement of dating and ruling, Messrs. Henry Bernd & Co. have perfected a physician's account book, whereby the indebtedness of a patient can be told at a glance and without having to "post the books," thereby saving the patient much time and labor, besides saving him many dollars in lost accounts during the year.

MISS FLORENCE NIGHTINGALE is said by an English publication to be a confirmed invalid from a spinal disease incurred through over-devotion to the cause of nursing. She went home sick from the Crimea, and her health was never thereafter thoroughly re-established. She is now approaching her seventieth year, and is destined to pass the evening of her days as an inmate, and its most favored inmate, of St. Thomas' Hospital, London. It was at that institution, in 1858, was established the Nightingale Fund of £50,000 in perpetual commemoration of the heroic labors of that lady in the Crimean hospitals.—*News*.

THE DOCTOR'S CODE—AN ETHICAL ODE.

Shall a doctor advertise?
Is it foolish? Is it wise?
Will it pay, or will it not?
"Yes, that trick will take the pot."

Since I think that it *will* pay
Comes the question—In what way?
Shall I start a monthly sheet,
Naming every fool I treat?

Shall I seek the public prints,
That at all deception squints?
Can I face the angry frowns
Of my rivals?—Stupid clowns!

No—a better way I see,
Wherewithal to catch the fee;
And my colleagues' eyes to "wool,"
With the celebrated "pull."

So if in the *Globe* I see
"The distinguished Dr. Bee
Called to Coventry to-day—
Gunshot wound—a street affray,"

How I cuss the whole *Globe* crew,
(To Dr. R. and P. and Q.)
And then down to newspaper row,
With many a smile and smirk, I go.

"You naughty man"—a sly side wink—
"You've broke the—Let's go take a drink."
. . . "Oh, Billy, by the way,
Was called to see Judge Cash to-day."

Another puff next week. And, too,
I've got another method—new!
It wouldn't do to sign a puff
In print—'twould raise a mighty huff.

But "word of mouth" dies in thin air;
You can't locate "talk" anywhere.
So wondrous cures of mine I tell,
Out-lying the manager of—well—

As thus: "Old Mrs. X.—bad case;
Giv'n up by five—cancer of the face;
My salve—one box; new creature now.
Case just like yours, cure you, I vow."

"Doc. Stokes? Too old. Clear out of date,
And Bacon—y-es, he'll have to wait;
Will *some* day make a *fair* M. D.,
But—swaddling-clothes—all theo*rie*."

Oh, selfish and short-sighted fool!
Didst ever read the "Golden Rule?"
Which teaches you should always do
As you would have men do to you.

—*J. W. M. in Indiana Medical Journal.*

THE Southern Surgical and Gynecological Association's postponed meeting will be held in the hall of the Young Men's Christian Association in Birmingham, Ala., on December 4, 5 and 6.

The programme which is given below is a good one and should attract a large attendance at the meeting, which we know will be both interesting and successful.

PROGRAMME OF THE ANNUAL MEETING.

TUESDAY—MORNING SESSION.

The Association will be called to order by the President at 10 o'clock a. m. 1. Prayer by the Rev. D. I. Purser, D. D. 2. Address of welcome by Hon. A. O. Lane. 3. Reading of minutes. 4. Report of the Committee of Arrangements. 5. Report of the Judicial Council. 6. Miscellaneous business.

The Annual Address of the President, Dr. W. D. Haggard, Nashville, Tenn.

Papers; 1. Gastrostomy. By W. B. Rogers, M. D., Memphis, Tenn. 2. Superinvolution of the Uterus following Trachelorraphy. By Virgil O. Hardon, M. D., Atlanta, Ga. 3. Indications for Operative Interference in Cerebral troubles. By T. O.

Summers, M. D., Jacksonville, Fla. 4. A Case of Tubal Pregnancy, presenting Interesting Medico-Legal Relations. By E. P. Sale, M. D., Memphis, Tenn. 5. The Extravagancies and Impractical Requirements of Modern Antiseptic Surgery, so far as the Country Practitioner is concerned. By J. M. Taylor, M. D., Corinth, Miss. 6. Antiseptics in Surgery and Gynecology. By F. T. Meriwether, M. D., Asheville, N. C. 7. My Antiseptic Bags; or, Practical Aseptic Surgery. By J. W. Long, Randleman, N. C.

*Adjournment at 2 p. m.*

Excursion to North Birmingham at 3:45 p. m.

EVENING SESSION AT 8 P. M.

The Annual Oration, by Dr. W. F. Hyer, of Meridian, Miss., at O'Brien's Opera House. After the oration the audience will be entertained by the Mendelssohn Club.

WEDNESDAY—MORNING SESSION AT 9:30 A. M.

1. Report of Treasurer. 2. Report of Judicial Council. 3. Miscellaneous business.

Papers: 8. Floating Kidney, with Vicarious Menstruation. By DeSaussure Ford, M. D., Augusta, Ga. 9. New Treatment of Intra-Uterine Fibroids. By E. J. Beall, M. D., Fort Worth, Texas. 10. The Medical Treatment of Fibroid Tumors of the Uterus. By Bedford Brown, M. D., Alexandria, Va. 11. Alexander's Operation. By W. L. Nichol, M. D., Nashville, Tenn. 12. The Present Status of Electro-Therapeutics in Gynecology. By J. R. Buist, M. D., Nashville, Tenn.

*Adjournment at 2 p. m.*

Excursion to Gate City at 3: 45 p. m.

EVENING SESSION AT 8 P. M.

13. Hysterectomy in Cancer of the Uterus. By W. H.

Wathen, M. D., Louisville, Ky. 14. Fractures of the Forearm. By Jno. Brownrigg, M. D., Columbus, Miss. 15. Treatment of Fractures with Plaster of Paris Splints. By W. F. Westmoreland, jr., M. D., Atlanta, Ga. 16. Interesting Cases of Surgery. By R. M. Cunningham, M. D., Pratt Mines, Ala. 17. The New Departure in Uterine Therapeutics—The Dry Method. By T. A. Means, M. D., Montgomery, Ala. 18. A Study of the Various Methods of Treatment of Laceration of the Perineum, and Rectocele, with Report of Cases. By J. H. Blanks, M. D., Meridian, Miss. 19. Perineal Lacerations. By M. C. Baldridge, M. D., Huntsville, Ala.

THURSDAY—MORNING SESSION AT 9:30 A. M.

1. Report of Judicial Council. 2. Unfinished and Miscellaneous Business.

Papers: 20. Cystoscopic Explorations. By A. V. L. Brokaw, M. D., St. Louis, Mo. 21. Shock of Injury and its Effects. By Jno. R. Page, M. D., Birmingham, Ala. 22. Some Practical Thoughts in Surgery and Gynecology. By Jas. Guild, M. D., Tuscaloosa, Ala. 23. The Field and Limitation of Laparotomy. By I. S. Stone, M. D., Lincoln, Va. 24. Operative Procedures in Traumatic Epilepsy. By J. T. Wilson, M. D., Sherman, Texas. 25. Surgical Tuberculosis. By W. Locke Chew, Birmingham, Alabama.

*Adjournment at* 1:30 *p. m.*

AFTERNOON SESSION AT 3 P. M.

26. Treatment of Certain Forms of Menorrhagia, with Report of Cases. By W. D. Bizzell, M. D., Atlanta, Ga. 27. Electrolysis in the Treatment of Urethral Strictures. By S. M. Hogan, M. D., Union Springs, Ala. 28. Operative Procedures in Hypertrophy of the Prostate. By R. D. Webb, M. D., Birmingham, Ala. 29. Electrolysis in Morbid Alterations produced in the Prostate by Gonorrhœa of the Urethra. By J. D. S. Davis, M. D., Birmingham, Ala. 30. Dermoid Cysts of the Coccygeal Region. By E. J. Beall, M. D., Fort Worth, Texas.

EVENING SESSION AT 8 P. M.

Discussion—Abdominal Surgery. Election and installation of officers.

GENERAL INFORMATION.—Members and visitors are expected to register. A room at the Y. M. C. A., corner Nineteenth Street and Second Avenue, will be provided for the purpose, from which the mail of the members and guests will be distributed, and at which the city residence of each member or guest can be ascertained.

Parties desiring to secure the excursion rate will closely observe the following rule, which will not be deviated from under any circumstances:

"No refund of fare will be made on any account whatever because of failure of the parties to obtain certificates."

You will observe from this rule that it will be absolutely necessary for each person to obtain a certificate from the agent when the ticket is purchased to the point where the convention is held; otherwise he will be unable to obtain the excursion rate returning, and will be obliged to pay full tariff fare both ways.

NOVEL TREATMENT FOR HEMORRHOIDS.—An amusing incident occurred a short time since in one of the northern counties of the State, whereby a granger accidentally discovered an efficient remedy for protrudng piles. It seems that "hog-killing" time was at hand, and the granger was cutting up and putting his meat into salt. He had just prepared the mixture with a plentiful supply of black and cayenne pepper, which he had thoroughly mixed together with his hands. Suffering badly from protruding piles, he went into the house and adjusted the offending members without washing his hands. Scarcely had he done so before the cayenne pepper took vigorous hold of the delicate parts, producing reflex contortions, which can be better imagined than described. He says, however, that the piles have not bothered him since, and he is prepared to bet that it will prove an efficacious remedy for the worst cases, if the patient has only the temerity to try it.—*Indiand Medical Journal.*

I QUESTION whether mankind in general is sufficiently impressed with the importance of strict cleanliness of the genital organs. Doctors should educate their patients to an appreciation of this matter, not only for esthetic reasons, but for healthful ones as well. The morning toilet should not be considered complete until a free evacuation of the bowels is secured, followed by a bathing of the anus and the entire genitalia.

Aside from the advantages in a cleanly and healthy direction, the moral and social well-being of the domestic circle would be subserved did our married women religiously cleanse the vagina by the use of free quantities of water, to which might well be added a slight modicum of common salt, borax or alum.

We know that the tooth-brush and cleanliness of the mouth prevent diseased gums and other disturbances of the cavity. How many cases of vaginitis, erosions of the os, balanitis and even urethritis may be chargeable to filthy, ill-kept vaginas may well be imagined.

Lawson Tait has said some things worthy of endorsement, among which is the following: "I am well satisfied that venereal diseases might be stamped out if more scrupulous attention were given to the toilet of the genitals."—*Review.*

DR. FRANK L. WYMAN, of Olneyville, Rhode Island, has been awarded $17,000 damages from the Union Horse Railroad Company for injuries received by being hurled from the front platform of a car which was driven at great speed in rounding a curve.

DR. HENRY B. SANDS, New York's leading surgeon, died suddenly November 18th, 1888.

IN January, 1889, there will be issued from the press of A. L. Chatterton & Co., New York, a new quarterly, entitled THE JOURNAL OF OPHTHALMOLOGY, OTOLOGY AND LARYNGOLOGY. It will be edited by Geo. S. Norton, M. D., assisted by Chas. Deady, M. D. Subscription price $3.00 per year. *The Journal* will be devoted to original articles upon the three specialties and made of the highest practical value to all interested in the eye, ear or throat. In addition to original papers by prominent authorities, the immense mass of material found at the N. Y. Ophthalmological Hospital will be utilized.

PAYMENTS ARE NECESSARY.—An exchange relates this parable: "A revivalist requested all in the congregation who paid their debts to rise. The rising was general. After they had taken their seats, a call was made for those who did not pay their debts, and one solitary individual arose and explained that he was an editor and could not pay because all the rest of the congregation were owing him their subscription to his paper."

IN commenting upon a suit for damages brought against a physician by the parents of a child which died after taking a mixture containing antipyrine and sweet spirits of nitre, which was prescribed by the physician, the *National Druggist*, November, 1888, declares that the crystalline deposit (isonitroso-antipyrine) produced by the union of these two agents is not poisonous. The editor says he has tested its toxicity upon himself as well as upon the lower animals.

A THIRTY DAYS' FAST.—Another trial of fasting is in progress at the Douglas Hotel, Edinburg. On this occasion, however, the alleged motive is the demonstration of the virtue of a herb, which the experimenter believes to be possessed of rare sustaining and nourishing properties. Alexander Jacques, the subject of the experiments, states that he will not part with the secret of the plant—which he learned from his grandmother—for a less sum than £20,000. In place of being used up at the end of the thirty days for which he has undertaken to fast, Jacques has challenged any man of his own weight to a boxing or wrestling contest. A strict watch is being exercised by a responsible vigilance committee. The fasting feat is a subject of much interest in medical circles in Edinburg, and M. Jacques' urine is being accurately observed and analyzed from day to day.—*British Medical Journal.*

SMELL OF SOUND MEAT.—The examination of the flesh of animals, from which the viscera have been removed, necessitates the analysis of all the tissues, the inspection of the fat, muscular tissue, fasciæ pleura and peritoneum, spinal cord, glands, vessels, blood, etc., before the meat can be accepted. In the normal state the flesh of every animal has its own characteristic odor. Beef has a special insipid kind of smell, modified by the different modes in which the animals have been fed. Thus it is stated that the flesh and milk of cattle in the polar regions have a fishy odor, because the absence of pasturage obliges the inhabitants to feed their oxen and cows on fish. Veal smells of milk, mutton of wool and sometimes grease. The normal odor of pork is insipid and inoffensive, but when the pigs are fed on offal the flesh has a pale cachectic hue, and an offensive smell and taste. The odor of poultry fed on corn differs from that of poultry artificially fattened. In a diseased state, meat emits a typical odor resembling the breath of feverish patients. This odor is particularly noticeable beneath the shoulder and in the muscles of the inner side of the leg. The odor should be carefully noted immediately after the incision is made. This should be done by the inspector himself. When diseased meat is roasted, it emits a strong and offensive smell. The fever odor is particularly marked in the case of animals which have suffered from peritonitis, charbon, morbid symptoms following parturition, or with ordinary acute disease. In such cases the smell is recognizable at once and it is unnecessary to make any incision. "Feverish" meat is always unfit for consumption on account of the leucomaines which it may contain. Moreover, there always exist pathological lesions which denote clearly that the animal was diseased before being killed.—*British Medical Journal.*

MR. KENNAN'S CHANGE OF VIEWS.—The following is an extract from a letter written home by Mr. Kennan while he was in Russia investigating the exile system for *The Century* magazine:

The exile system is much worse than I supposed. Mr. ——'s examination of prisons and study of the exile system were extremely superficial. I cannot understand how, if he really went through the Tiumen and Tomsk forwarding prisons, he could have failed to see that their condition and the condition of their wretched inmates were in many respects shocking. Nobody here has tried to conceal it from me. The acting governor of this province said to me very frankly yesterday that the condition of the Tomsk prison is "oozhasnoi" (awful), but that he cannot help it. . . . What I have previously written and said about the treatment of the political exiles seems to be substantially true and accurate—at least so far as Western Siberia is concerned, but my preconceived ideas as to their character have been rudely shaken. The Russian liberal and revolutionists whom I have met here are by no means half-educated enthusiasts, crazy fanatics or men whose mental processes it is difficult to understand. On the contrary, they are simple, natural, perfectly comprehensible and often singularly interesting and attractive. One sees at once that they are educated, reasonable, self-controlled gentlemen, not different in any essential respect from one's self.

When I write up this country for *The Century*, I shall have to take back some of the things that I have said. The exile system is worse than Ibelieved it to be, and worse than I have described it. It isn't pleasant, of course, to have to admit that one has written upon a subject without fully understanding it; but even that is better than trying, for the sake of consistency, to maintain a position after one sees that it is utterly untenable.

PROFESSOR OF MATERIA MEDICA (lecturing on tannin).—"And by the by, gentlemen, tannic acid is the antidote to the poison of the mushroom; can any of you explain its action?"

Texas Student—"T-t-think I can, professor!"

"Well, sir, explain to the class the chemical reactions that occur and how tannin acts as antidote to the poison of the poisonous mushroom."

"It f-f-forms the t-t-tannate of m-mush, and leaves room in the s-s-stomach."—*Texas Medical Journal.*

Preparatory to having a tooth extracted, a Dennison, Tex as, negro took laughing gas. The effect was startling, for he leaped through a window carrying sash and all with him to the ground, 20 feet below. His surprise on recovering consciousness far exceeded his injuries.

AN EXCHANGE refers to the fact that the 2,476 physicians of New York city are said to agree that "the profession is overcrowded," and the same page contains the statement that in Tomsk, Siberia, there are only twenty-two doctors, an average in some districts of only one physician to 100,000 inhabitants. What a blissful region for an unoccupied medical man to emigrate to! Only imagine an idle doctor suddenly possessed of a clientile of 100,000 people!

By the way, wouldn't it be a good plan for one-half of the over-crowded profession of New York city to chip in and ship the other half to the elysian fields of Siberia?—*Review.*

